# Exploring Vitamin D Trends Through Big Data Analysis

**DOI:** 10.3390/nu17233808

**Published:** 2025-12-04

**Authors:** Szilvia Racz, Miklos Emri, Ervin Berenyi, Laszlo Horvath, Bela E. Toth, Sandor Barat, Edit Kalina, Luca Jozsa, Amrit Pal Bhattoa-Buzas, William B. Grant, Harjit Pal Bhattoa

**Affiliations:** 1 Department of Medical Imaging, Faculty of Medicine, Kalman Laki Doctoral School of the University of Debrecen, University of Debrecen, 4032 Debrecen, Hungary; lakatos.szilvia@med.unideb.hu; 2 Department of Medical Imaging, Faculty of Medicine, University of Debrecen, 4032 Debrecen, Hungary; emri.miklos@med.unideb.hu (M.E.); eberenyi@med.unideb.hu (E.B.); 3 Department of Pharmaceutical Surveillance and Economics, Faculty of Pharmacy, University of Debrecen, 4032 Debrecen, Hungary; horvath.laszlo@pharm.unideb.hu (L.H.); tothe.bela@pharm.unideb.hu (B.E.T.); 4 Department of Laboratory Medicine, Faculty of Medicine, University of Debrecen, 4032 Debrecen, Hungary; barath.sandor@med.unideb.hu (S.B.); ekalina@med.unideb.hu (E.K.);; 5 Sunlight, Nutrition, and Health Research Center, 1745 Pacific Ave., Suite 504, San Francisco, CA 94109, USA; wbgrant@infionline.net

**Keywords:** 25-hydroxyvitamin D, big data, methodology, seasonal variations, age, gender, vitamin D

## Abstract

Background/Objectives: Big data analysis has revolutionized medical research, making it possible to analyze vast amounts of data and gain valuable insights that were previously impossible to obtain. Our knowledge of the characteristics of vitamin D sufficiency is primarily based on data from a limited number of observations, generally spanning a few years at most. Methods: Here at the Medical Faculty of the University of Debrecen, the big data approach has allowed us to analyze trends in vitamin D status using nearly 60,000 25-hydroxyvitamin D (25(OH)D) concentration results from 2000 onwards. Results: Apart from analyzing the well-known phenomenon of seasonality in 25(OH)D concentration, we observed a trend in test requests, which increased from a few hundred in 2000 to almost 10,000 in 2020. Of particular interest is the change in the gender gap in test requests. In previous years, test requests were primarily from women, but by the end of the analysis period, a significant number of requests were from men as well. Since the data set includes all age groups, we analyzed 25(OH)D concentration for incremental age sets of five years, from a few months to 100 years old. The prevalence of vitamin D insufficiency (<75 nmol/L) was clearly demarcated among various years of observation, age groups, sexes, and seasons. Our data was particularly valuable for analyzing the effect of the methodology used for 25(OH)D determination. Three different methodologies were used during the study period, and clear, statistically significant bias was observed. Conclusions: Our results clearly demonstrate the effect of the methodology used to determine 25(OH)D concentrations on vitamin D status, explicitly highlighting the urgent need to standardize the various platforms used to measure this important analyte and its consequences for public health.

## 1. Introduction

The concept of big data in nutritional epidemiology has gained traction, with numerous large-scale prospective studies accumulating over the past 50 years [1]. While traditional randomized controlled trials (RCTs) are considered the gold standard for intervention studies, recent large-scale vitamin D3 RCTs have not yielded significant primary results [2]. A recent critical appraisal indicates that the reported outcomes and methodological constraints of these RCTs should guide the design of future vitamin D and nutrition interventions, emphasizing the need for a more individualized or precision-based approach [3]. An alternative approach involves segregating participants based on their responsiveness to vitamin D3 supplementation and measuring genome-wide parameters over multiple time points

Studies comparing vitamin D data across different years have yielded mixed results. A population-based study in northern Sweden found no clear trend in 25-hydroxyvitamin D 25(OH)D concentrations between 1986 and 2014 [4]. However, an analysis of US population data revealed lower serum 25(OH)D concentrations in 2000–2004 compared to 1988–1994, partly due to assay changes and factors like increased body mass index and sun protection [5]. The reliability of single 25(OH)D measurements over time has been examined, with one study finding a moderate correlation (r = 0.75) between two measurements taken within a year [6]. These studies highlight the importance of considering methodological factors when comparing vitamin D data across different time periods.

Seasonal variation in 25(OH)D concentrations is well-documented, with the lowest concentrations typically observed in winter and the highest in summer/autumn [7,8,9]. This seasonality can significantly impact health risk assessments and clinical decision-making [7]. Studies have shown that vitamin D deficiency prevalence can be approximately double in winter/spring compared to summer/autumn [8,9,10,11]. Researchers emphasize the importance of considering blood sampling seasonality as a crucial preanalytical factor in vitamin D assessment [8,9].

Lippi et al. reported no significant differences in 25(OH)D concentrations or deficiency prevalence across age groups or genders in their study conducted in north-east Italy [12]. Malyavskaya et al. observed high frequencies of vitamin D deficiency in all age groups in Arkhangelsk, Russia, with the highest prevalence being in adolescents and adults [13]. Yeşiltepe-Mutlu et al. found that children under 3 years old in Turkey had significantly higher vitamin D levels compared to other age groups, likely due to a national supplementation program [14]. They also noted lower levels in adults, suggesting a need for supplementation in older populations [14]. These findings highlight the complexity of vitamin D status across populations and emphasize the importance of targeted interventions.

The present study aimed to draw temporal inferences on gender disparity, age groups referred, seasonal variability and methodology of analyses, using a big data approach in the framework of a single regional medical healthcare provider.

## 2. Materials and Results

Anonymized medical data from the year 2000 onwards is available for research purposes at the Medical Faculty of the University of Debrecen. Anonymization is assured using the pseudonymization system of the UDBD Health data warehouse, in accordance with University of Debrecen data warehouse regulations. This Microsoft Azure Cloud-based UDBD-Health database was used to retrieve all 25(OH)D measurement data between 12 January 2000 and 31 December 2020. At the University of Debrecen, the Institutional Research Ethics Committee has permitted the use of anonymized data generated during standard healthcare for research purposes (Permission number—DE RKEB/IKEB 5404-2020 issued on 13 January 2020). No exclusion criteria were applied. Apart from the 25(OH)D concentrations, the date of the measurement, sex and the age of the patient were also retrieved. Information about the date when the methodology used to measure 25(OH)D was changed was available from the laboratory. 25(OH)D concentrations <75 nmol/L and ≥75 nmol/L were coded as insufficient and sufficient, respectively. The in-house developed R (R version 4.2.2; R Core Team, 2022) statistical program was used for the statistical analysis. Descriptive statistical summaries, FDR correction and a Wilcoxon rank-sum test were performed. Figures were generated using the R (version 4.2.2) software. A value of *p* < 0.05 was considered statistically significant.

**Figure 1:**
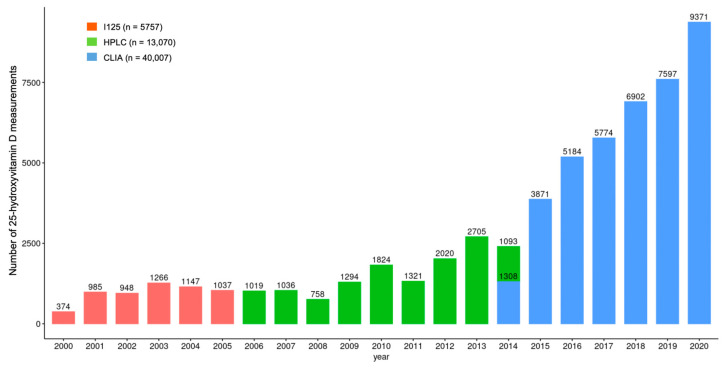
Differences in the number of 25-hydroxyvitamin D measurements over the study period. I^125^: Radioimmunoassay; HPLC: High-pressure liquid chromatography; CLIA: Chemiluminescence immunoassay.

As part of routine healthcare, a total of 58,834 test results were identified that belonged to 31,710 patients (22,447 women and 9263 men). The number of tests per year showed an increasing tendency over time. The number of tests conducted in 2020 was 25 times that in 2001 (Figure 1). During the study period, three different analytical methods, a radioimmunoassay (RIA) (before 30 January 2006 (n = 5757)), high-pressure liquid chromatography (HPLC) (between 30 January 2006 and 16 June 2014 (n = 13,070)) and a chemiluminescence immunoassay (CLIA) (from 16 June 2014 (n = 40,007)), were used for 25(OH)D determination (Figure 1.). The inter-assay CV for the I^125^ radioimmunoassay (Diasorin Inc., Stillwater, MN, USA), high-pressure liquid chromatography (HPLC) using a Jasco HPLC system (Jasco, Tokyo, Japan) and Bio-Rad reagent kit (Bio-Rad Laboratories, Hercules, CA, USA), and the automated Liaison DiaSorin total 25(OH)D chemiluminescence immunoassay (CLIA) (DiaSorin Inc., Stillwater, MN, USA) was <13%, <3.5% and <7.8%, respectively.

**Figure 2:**
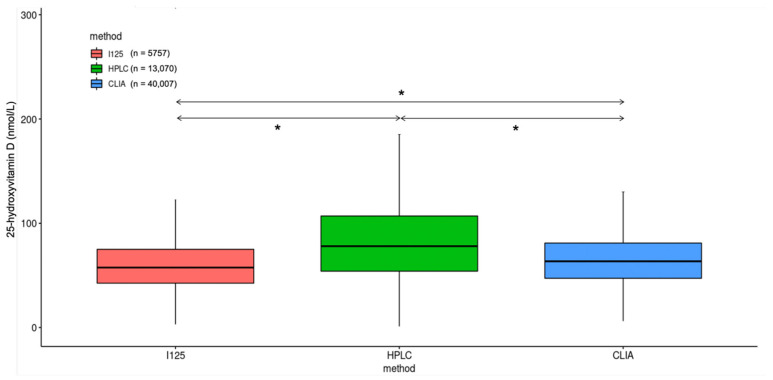
Difference in measured 25-hydroxyvitamin D concentrations using radioimmunoassay (I^125^), high-pressure liquid chromatography (HPLC) and chemiluminescence immunoassay (CLIA). * *p* < 0.001.

There was a statistically significant difference between the average 25(OH)D concentrations when comparing the test results measured on the different analytical platforms, particularly concentrations measured with HPLC being higher than with RIA and CLIA (Figure 2).

**Figure 3:**
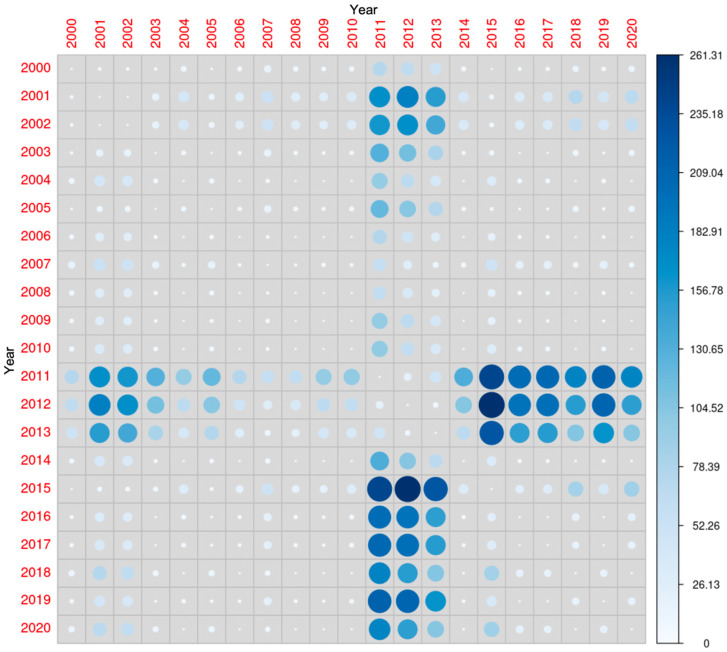
Comparison of 25-hydroxyvitamin D concentrations during the study period. The diameter of each circle and the intensity of its color shading denote the magnitude of disparity between the years represented along the x- and y-axes. Larger diameters and darker color tones correspond to a greater degree of difference between the two years under comparison.

Although 25(OH)D concentrations were significantly different when comparing multiple years, the average concentrations were the highest in the years 2011, 2012 and 2013 (Figure 3).

**Figure 4:**
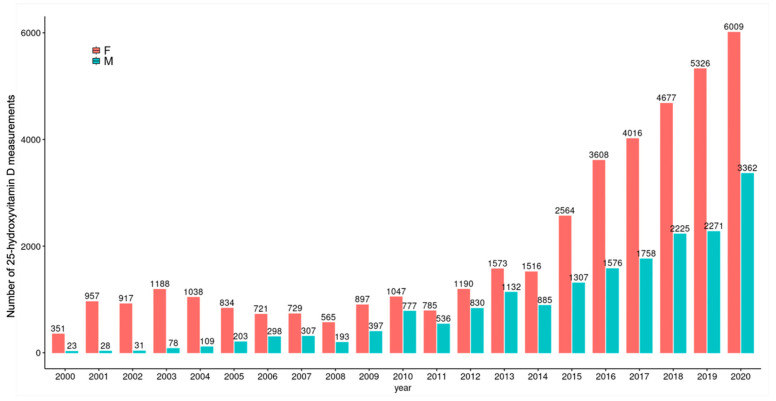
Frequency of testing by gender. F: Female, M: Male.

In the study period, there was a gradual increase in the number of tests per year for both sexes, an increase of 6 (n = 957 in 2001, n = 6009 in 2020) and 120 (n = 28 in 2001, n = 3362 in 2020) times was observed for women and men, respectively. Even though there was an increase in testing for men, women were still tested twice as frequently as men in 2020. In 2001, 97% of the results were from women and only 3% from men; this changed to 64% in women and 36% in men in 2020 (Figure 4).

**Figure 5:**
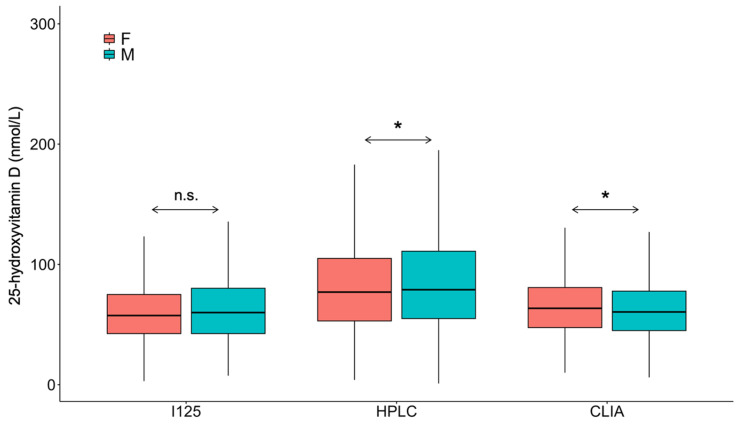
Overall gender difference in 25-hydroxyvitamin D levels. F: Female; M: Male; I^125^: Radioimmunoassay; HPLC: High-pressure liquid chromatography; CLIA: Chemiluminescence immunoassay. * *p* < 0.001. n.s.: No significance.

**Figure 6:**
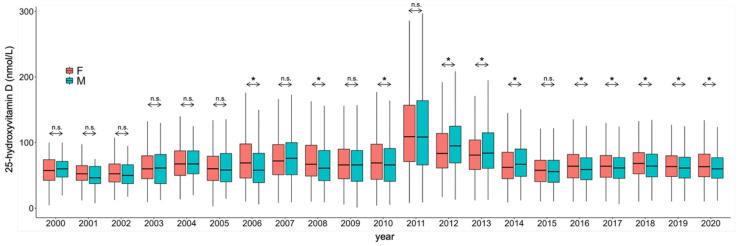
Gender differences in 25-hydroxyvitamin D concentrations during the study period. F: Female; M: Male. * *p* < 0.001. n.s.: No significance.

Although, when comparing average concentrations during the study period, there was no difference in 25(OH)D concentrations when comparing the two sexes (69 ± 7 nmol/L (women) vs. 70 ± 8 nmol/L (men); *p* > 0.05), men had significantly higher average concentrations when determinations were carried out with the HPLC methodology (Figure 5) and there were several years when there was a statistically significant difference between the two sexes (Figure 6).

Over the period when the RIA methodology was in use, no significance was found when comparing the two sexes. When using HPLC, in 2006, 2008 and 2010, women had higher averages, and in 2012, 2013 and 2014, men were higher. Except for the year 2015 (where women had non-significantly higher values), in all the other years where the CLIA methodology was used, women had significantly higher vitamin D concentrations.

During the overall study period, there was no significant change in the percentage of vitamin D-sufficient patients (Table 1). Hypovitaminosis D was defined as 25(OH)D levels <75 nmol/L, as suggested by Dawson-Hughes et al. [15].

**Figure 7:**
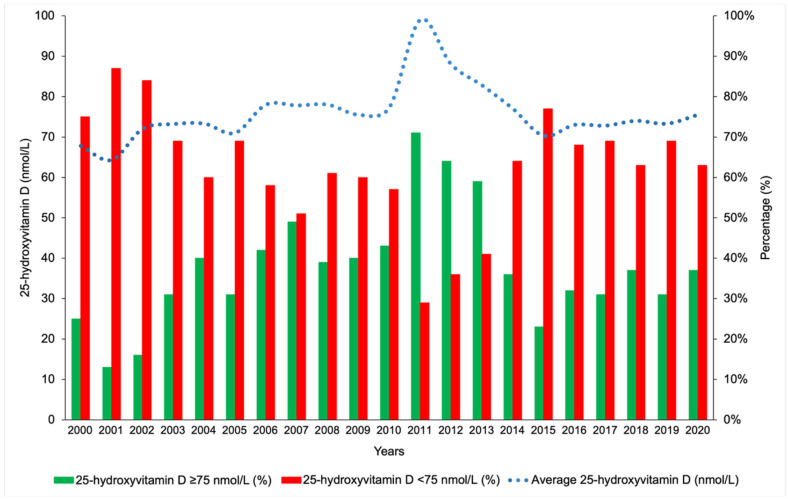
Trend of 25-hydroxyvitamin D concentrations during the study period.

It was particularly interesting to note that within the study period spanning between 2000 and 2020, the average 25(OH)D concentrations and the percentage of sufficient vitamin D status were the highest during the years 2011, 2012 and 2013 when the methodology used was HPLC (Figure 7).

**Figure 8:**
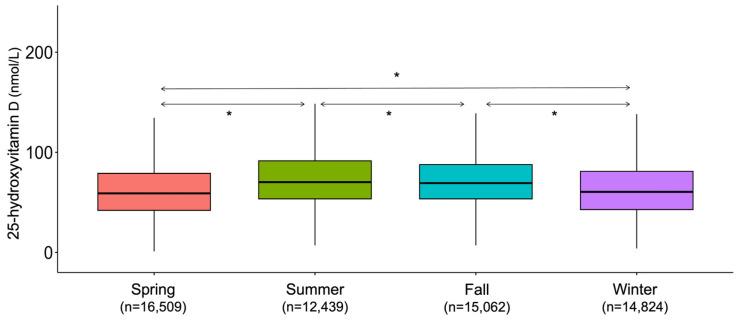
Seasonality trends during the study period. * *p* < 0.001.

Seasonal variation was pronounced during the study period (Figure 8).

**Figure 9:**
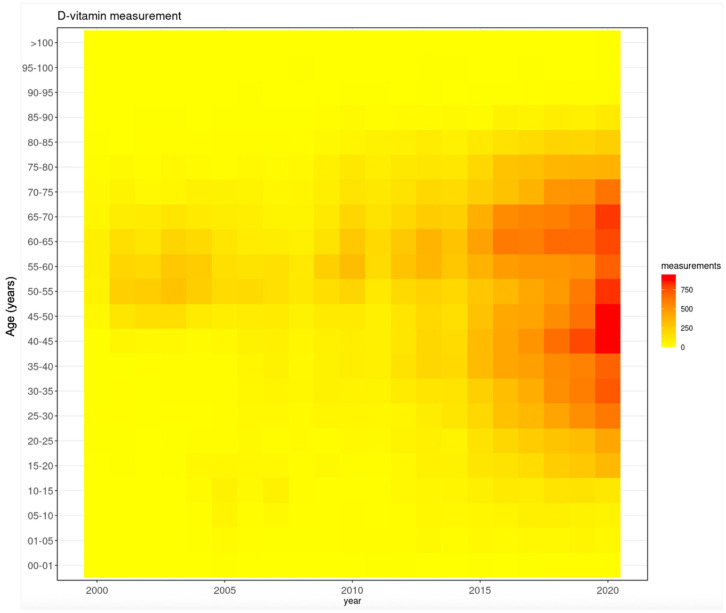
Trends in testing frequency in the various age groups during the study period.

In the early 2000s, the majority of tests were conducted on those between 45 and 65 years of age. From 2010 onwards, this age range widened to 10–85 years, and the highest number of tests was in those from 40 to 45 and 60 to 65 years (Figure 9).

**Figure 10:**
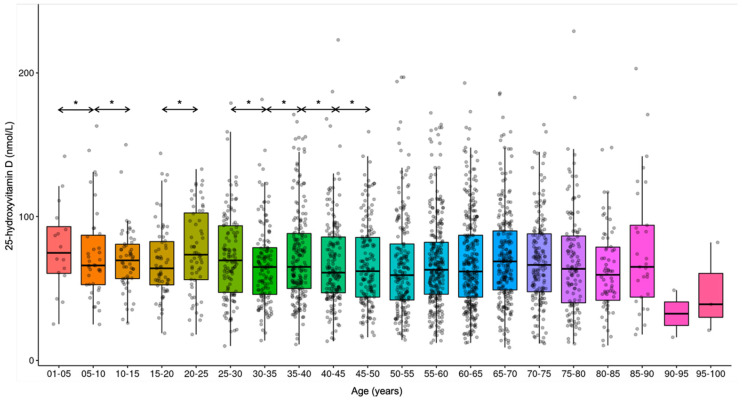
Trends in 25-hydroxyvitamin D concentrations in the various age groups. * *p* < 0.001.

**Figure 11:**
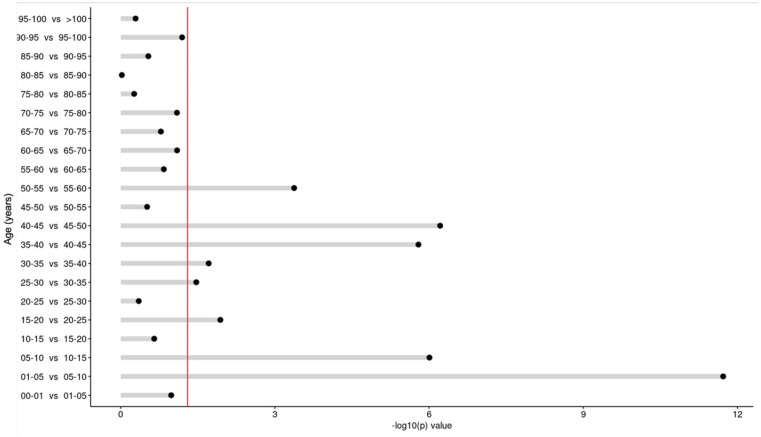
Difference in vitamin D concentrations in sequential comparison of the age groups. The red line depicts the statistically significant value limit.

Comparing the age groups (years) sequentially, significant differences were noticed in the average vitamin D concentrations: 0–1 yrs vs. 1–5 yrs (1–5 yrs higher; *p* < 0.001), 1–5 yrs vs. 5–10 yrs (5–10 yrs lower; *p* < 0.001), 5–10 yrs vs. 10–15 yrs (10–15 yrs higher; *p* < 0.001), 15–20 yrs vs. 20–25 yrs (20–25 yrs higher; *p* < 0.001), 25–30 yrs vs. 30–35 yrs (30–35 yrs lower; *p* < 0.001), 30–35 yrs vs. 35–40 yrs (35–40 yrs higher; *p* < 0.001), 35–40 yrs vs. 40–45 yrs (40–45 yrs lower; *p* < 0.001), 40–45 yrs vs. 45–50 yrs (45–50 yrs higher; *p* < 0.001). (Figure 10 and Figure 11).

**Figure 12:**
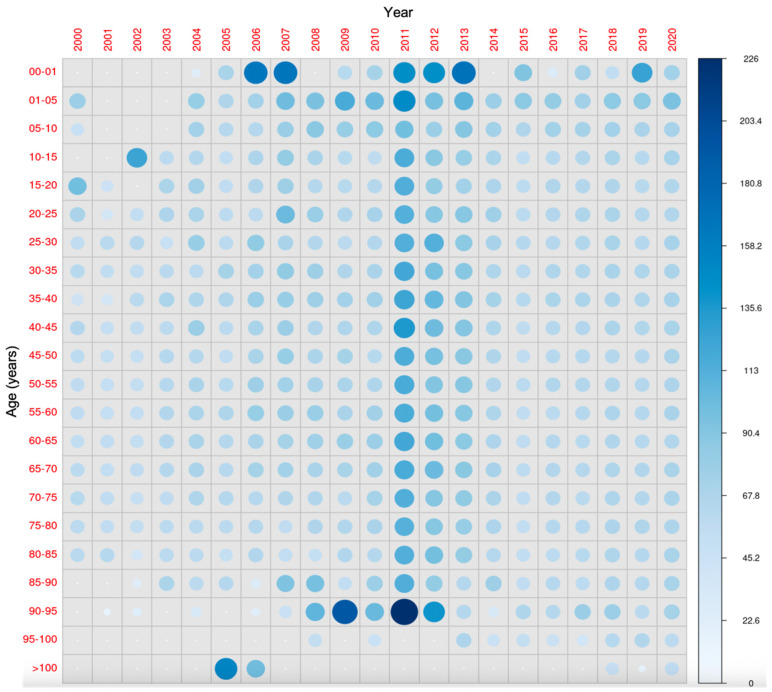
All-age-groups comparison of 25-hydroxyvitamin D concentrations during the study period. The diameter of each circle and the intensity of its color shading denote the magnitude of the disparity between the years (x-axis) and age groups (y-axis). Larger diameters and darker color tones correspond to a greater degree of difference between the two years (for the same age group) and age groups (in the same year) under comparison.

Figure 12 shows the comparison across all age groups during the study period. It is observed that after the early years, when vitamin D supplementation is compulsory, the concentrations tend to decrease in the later years of childhood. Such changes are not evident in the adult age groups.

## 3. Discussion

In recent years, researchers have leveraged the power of large-scale data analysis to uncover valuable insights into the prevalence and patterns of vitamin D sufficiency and insufficiency across diverse populations (Table 2) [4,5,16,17,18,19,20,21,22,23,24,25,26,27,28,29].

One key finding from this body of research is the estimation of the percentage of individuals with sufficient versus insufficient 25(OH)D concentrations. Numerous large-scale studies have consistently reported that a significant proportion of the global population, often exceeding 50%, exhibits suboptimal vitamin D status. This widespread prevalence of vitamin D insufficiency is a major public health concern, as inadequate 25(OH)D concentrations have been linked to a range of adverse health outcomes, including increased risks of bone loss, cardiovascular disease, many types of cancer and other chronic conditions [30,31,32]. The sheer scale of this problem underscores the critical need for targeted interventions and public health strategies to address this pervasive issue and improve overall vitamin D status within the population.

Furthermore, researchers have explored the temporal trends of 25(OH)D levels, conducting comparative analyses across multiple years. These in-depth investigations have unveiled valuable insights into the intricate interplay between vitamin D status and various demographic factors, such as seasonal variations, age, and gender [33,34]. The findings from these studies suggest that 25(OH)D levels may exhibit distinct patterns among different population subgroups, underscoring the need for a more nuanced understanding of this complex relationship [34].

A deeper examination of the big data on 25(OH)D has revealed significant differences in vitamin D status between men and women, as well as across different age groups. Some studies have found that older adults and women tend to have lower 25(OH)D concentrations compared to younger individuals and men, respectively [25]. These observed disparities highlight the importance of developing targeted public health interventions and educational campaigns to address the specific needs of these population subgroups. By tailoring strategies to address the unique vitamin D-related challenges faced by different demographics, healthcare providers and policymakers can work towards more effective and equitable solutions to improve overall vitamin D sufficiency within the broader population.

The determination of circulating 25(OH)D concentrations is recommended solely for patients deemed to be at risk of vitamin D deficiency by current guidelines, where the different risk factors are exhaustively enumerated [35,36].

Being the most abundant vitamin D metabolite in the bloodstream, 25(OH)D is considered the most optimal marker of vitamin D status. The current evidence points to 25(OH)D levels showing significant association with clinical endpoints, e.g., bone mineral density, fracture risk, falls, numerous pleotropic effects and mortality [25,37,38,39,40,41,42]. Having a relatively long half-life of 2–3 weeks, serum levels rarely oscillate within a short period; furthermore, 25(OH)D reflects the amount of both intake and production [43]. Additionally, 25(OH)D levels change in accordance with sunlight (UV) exposure and vitamin D supplementation [38,44,45,46,47,48,49,50,51].

Although considered an optimal metabolite reflecting vitamin D status, the determination of 25(OH)D concentrations is still challenging despite recent technological improvements [52]. It is a prerequisite that 25(OH)D assays should measure both 25(OH)D_2_ and 25(OH)D_3_, i.e., total 25(OH)D. Adding to the technical challenges is the strongly hydrophobic nature of 25(OH)D that circulates in bound form with vitamin D-binding protein (DBP), albumin and lipoproteins; as such, stripping 25(OH)D from its carriers is a preliminary step in the analytics of total 25(OH)D concentrations [53].

Adding further to the technical difficulties is the fact that 25(OH)D_2_ and 25(OH)D_3_ have non-similar affinity constants for their carrier proteins; as such, ideal dissociation is a prerequisite for the precise quantification of total 25(OH)D levels. Since there is no room for organic solvent extraction, this can be a limiting step in automated immunoassays, in contrast to manual radioimmunoassays and chromatographic and binding protein assays. Alternative releasing agents are usually used in automated immunoassays that do not always achieve the desired dissociation. Additionally, due to the above-mentioned technical lapses, determination with automated methodology is usually less reliable when DBP levels are elevated, e.g., in pregnancy, estrogen therapy or renal failure [54,55,56,57].

Although ample data on 25(OH)D concentrations exist, standardizing 25(OH)D values remains challenging. To address this issue, the Vitamin D Standardization Program (VDSP) has introduced protocols for standardizing existing 25(OH)D data from national surveys around the world [17,58,59,60,61]. In brief, the VDSP protocol involves identifying a batch of samples from the sample pool primarily used to determine 25(OH)D in the given survey. These samples are then reanalyzed using the reference measurement procedures (RMP) of the National Institute of Standards and Technology (NIST) and Ghent University. The results are then used to correct the initially measured 25(OH)D concentrations of the sample pool. While the protocol is well-defined, the recommended RMP requires specialized equipment and expertise primarily available at specialized laboratories. Furthermore, the financial aspect may be a limitation. Nonetheless, Jakab et al. have proposed correcting the measured 25(OH)D values using the linear regression bias from the NIST “total” target values reported by the Vitamin D External Quality Assessment Scheme (DEQAS) [62].

All clinical and research laboratories are strongly encouraged to participate in accuracy-based external quality assessment programs, such as those offered by the College of American Pathologists (CAP) or DEQAS. Providers of these programs should conduct regular commutability studies to ensure that their reported results are consistent with clinical outcomes obtained across various assay methods. Furthermore, manufacturers are also encouraged to participate in the Vitamin D Standardization-Certification Program (VDSCP).

Significant methodological progress has occurred in the measurement of 25(OH)D and its metabolites. A recent review comprehensively evaluated the capabilities of available commercial assay platforms [63]. LC–MS/MS continues to be recognized as the definitive method for the accurate quantification of clinically relevant, circulating vitamin D metabolites.

## 4. Limitations

The data analyzed is taken from the database of the University of Debrecen. Although it is one of the biggest healthcare providers in the country, it caters mainly to the needs of the population residing in the northern region of Hungary. Furthermore, the data presented inherently belong to the healthcare setting and do not represent a population-based survey. As such, the deductions of our study may not be readily applicable to the whole population. Furthermore, analyses of the result categories of <50 nmol/L and <25 nmol/L were not performed.

## 5. Conclusions

Our results clearly demarcate the effect of the methodology used to determine 25(OH)D vitamin concentrations on Vitamin D status, explicitly highlighting the urgency of the standardization of various platforms used to measure this ominous analyte with grave public health importance and, therefore, consequences.

## Figures and Tables

**Table 1:** Gender distribution of the vitamin D insufficient and sufficient cases in each year.

Year	Sex	Vitamin D-Insufficient (<75 nmol/L) Cases		Vitamin D-Sufficient (≥75 nmol/L) Cases	
		N	25-Hydroxyvitamin D (nmol/L) (Mean, Range)	N	25-Hydroxyvitamin D (nmol/L) (Mean, Range)
2000	F	263 (70.3%)	48 (4–73)	88 (23.5%)	86 (75–100)
	M	17 (4.5%)	52 (19–68)	6 (1.6%)	85 (75–100)
2001	F	827 (84.0%)	49 (12–74)	130 (13.2%)	86 (75–125)
	M	26 (2.6%)	47 (7–72)	2 (0.2%)	75 (75–75)
2002	F	767 (80.9%)	47 (12–72)	150 (15.8%)	90 (75–150)
	M	27 (2.8%)	47 (17–72)	4 (0.4%)	104 (80–125)
2003	F	822 (64.9%)	50 (9–72)	366 (28.9%)	94 (75–150)
	M	53 (4.2%)	45 (12–72)	25 (2.0%)	103.9 (77–200)
2004	F	623 (54.3%)	51 (14–72)	415 (36.2%)	96 (75–162)
	M	63 (5.5%)	52 (20–72)	46 (4.0%)	94 (75–125)
2005	F	582 (56.1%)	48 (3–75)	252 (24.3%)	94 (75–182)
	M	136 (13.1%)	46 (14–75)	67 (6.5%)	96 (75–158)
2006	F	391 (38.4%)	47 (10–74)	330 (32.4%)	110 (75–295)
	M	199 (19.5%)	44 (6–74)	99 (9.7%)	111 (75–260)
2007	F	385 (37.2%)	49 (8–74)	344 (33.2%)	108 (75–248)
	M	145 (14.0%)	47 (9–74)	162 (15.6%)	107 (75–241)
2008	F	335 (44.2%)	51 (10–74)	230 (30.3%)	111 (75–280)
	M	124 (16.4%)	47 (9–74)	69 (9.1%)	103 (76–195)
2009	F	541 (41.8%)	48 (6–74)	356 (27.5%)	107 (75–267)
	M	238 (18.4%)	45 (1–74)	159 (12.3%)	102 (75–211)
2010	F	576 (31.6%)	45 (4–74)	471 (25.8%)	110 (75–293)
	M	463 (25.4%)	45 (5–74)	314 (17.2%)	109 (75–287)
2011	F	222 (16.8%)	51 (8–74)	563 (42.6%)	144 (75–296)
	M	161 (12.2%)	50 (9–74)	375 (28.4%)	151 (75–297)
2012	F	474 (23.5%)	55 (17–74)	716 (35.4%)	116 (75–292)
	M	251 (12.4%)	56 (13–74)	579 (28.7%)	125 (75–300)
2013	F	678 (25.1%)	54 (12–74)	895 (33.1%)	109 (75–258)
	M	443 (16.4%)	53 (13–74)	689 (25.5%)	115 (75–283)
2014	F	989 (41.2%)	49 (9–75)	527 (21.9%)	103 (75–262)
	M	544 (22.7%)	52 (12–75)	341 (14.2%)	104 (75–229)
2015	F	1976 (51.0%)	49 (10–75)	588 (15.2%)	93 (75–233)
	M	1003 (25.9%)	48 (10–75)	304 (7.8%)	91 (75–208)
2016	F	2404 (46.4%)	51 (10–75)	1204 (23.2%)	96 (75–290)
	M	1147 (22.1%)	50 (10–75)	429 8.3%)	95 (75–229)
2017	F	2697 (46.7%)	51 (10–75)	1319 (22.8%)	95 (75–287)
	M	1262 (21.9%)	51 (6–75)	496 (8.6%)	94 (75–255)
2018	F	2899 (42.0%)	54 (10–75)	1778 (25.8%)	95 (75–262)
	M	1476 (21.4%)	52 (12–75)	749 (10.8%)	95 (75–214)
2019	F	3597 (47.3%)	52 (10–75)	1729 (22.8%)	94 (75–240)
	M	1626 (21.4%)	51 (11–75)	645 (8.5%)	96 (75–265)
2020	F	3639 (38.8%)	52 (10–75)	2370 (25.3%)	99 (75–272)
	M	2220 (23.7%)	52 (11–75)	1142 (12.2%)	99 (75–246)

**Table 2:** Big data studies on Vitamin D.

Study	Year of Publication	Country	Study Period	Age	Gender (Women%:Men%)	N	Method
Looker et al. [5]	2008	USA	1988–1994 and 2000–2004	20–59 years		18,158 + 20,289	Radioimmunoassay
Hintzpeter et al. [21]	2008	Germany	October 1997–March 1999	18–79 years	56.3:43.7	4,030	CLIA
Ginde et al. [25]	2009	USA	1988–1994 and 2000–2004	≥12 years	52.4:47.6	18,883 + 13,369	Radioimmunoassay
Nielsen et al. [24]	2014	Greenland	1987 and 2005–2010	≥18 years	56.6:43.4	306 + 2877	LC-MS/MS
McKenna et al. [23]	2015	Ireland	1993–2013	birth–105 years	66.7:32.3	43,782	Hadad and Chyu competitive radioimmunoassay (1974–1994), Incstar/Diasorin radioimmunoassay (1994–2008), Immundiagnostic Systems radioimmunoassay (2008–2011), Elecsys Vitamin D Total (2011-)
Sarafin et al. [17]	2015	Canada	2007–2011	3–79 years	52:48	5306 + 6030	CLIA
Hoge et al. [20]	2015	Wallonia (Belgium)	May 2010–March 2012	20–69 years	51.3:48.7	915	CLIA
Schleicher et al. [18]	2016	USA	2007–2010	≥1 year	50:50	15,650	LC-MS/MS
Rabenberg et al. [19]	2018	Germany	1998–2011	1–79 years	50:50	20,927	Originally CLIA, reanalyzed LC-MS/MS
Kunz et al. [27]	2019	Germany	2009–2014	1–17 years	46.9:53.1	1929	CLIA
Herrick et al. [26]	2019	USA	2011–2014	≥1 year	50.5:49.5	16,180	Radioimmunoassay
Petrenya et al. [22]	2019	Northern Norway	2012–2014	40–69 years	54.3:45.7	4465	Immunoassay (IDS-iSYS)
Summerhays et al. [4]	2020	Northern Sweden	1986–2014	25–74 years	51:49	11,129	One-step immunoassay
Tuuminen et al. [29]	2022	Finland	1987–2020		68:32	67,236	HPLC
Smirnova et al. [16]	2022	Russia	2013–2018	≥18 years	83:17	30,040	Chemiluminescent Microparticle Assay
Horváth et al. [28]	2023	Hungary	1 January 2015–30 June 2021	birth–100 years	68.4:31.6	45,567	CLIA

## Data Availability

The data supporting the findings of this study are available from the corresponding author upon reasonable request. The data are not publicly available due to privacy restrictions.

## Abbreviations

The following abbreviations are used in this manuscript:

25(OH)D	25-hydroxyvitamin D
CAP	College of American Pathologists
CLIA	Chemiluminescence immunoassay
DBP	Vitamin D binding protein
DEQAS	Vitamin D External Quality Assessment Scheme
F	Female
HPLC	High-pressure liquid chromatography
LC-MS/MS	Liquid chromatography mass spectrometry/mass spectrometry
M	Male
NIST	National Institute of Standards and Technology
RCT	Randomized controlled trials
RIA	Radioimmunoassay
RMP	Reference measurement procedures
VDSP	Vitamin D Standardization Program
VDSCP	Vitamin D Standardization-Certification Program

## References

[1] Satija A., Hu F.B. (2014). Big data and systematic reviews in nutritional epidemiology. Nutr. Rev..

[2] Gospodarska E., Ghosh Dastidar R., Carlberg C. (2023). Intervention Approaches in Studying the Response to Vitamin D_3_ Supplementation. Nutrients.

[3] Pilz S., Trummer C., Theiler-Schwetz V., Grubler M.R., Verheyen N.D., Odler B., Karras S.N., Zittermann A., Marz W. (2022). Critical Appraisal of Large Vitamin D Randomized Controlled Trials. Nutrients.

[4] Summerhays E., Eliasson M., Lundqvist R., Soderberg S., Zeller T., Oskarsson V. (2020). Time trends of vitamin D concentrations in northern Sweden between 1986 and 2014: A population-based cross-sectional study. Eur. J. Nutr..

[5] Looker A.C., Pfeiffer C.M., Lacher D.A., Schleicher R.L., Picciano M.F., Yetley E.A. (2008). Serum 25-hydroxyvitamin D status of the US population: 1988–1994 compared with 2000–2004. Am. J. Clin. Nutr..

[6] Major J.M., Graubard B.I., Dodd K.W., Iwan A., Alexander B.H., Linet M.S., Freedman D.M. (2013). Variability and reproducibility of circulating vitamin D in a nationwide U.S. population. J. Clin. Endocrinol. Metab..

[7] Rosecrans R., Dohnal J.C. (2014). Seasonal vitamin D changes and the impact on health risk assessment. Clin. Biochem..

[8] Bonelli P., Buonocore R., Aloe R., Lippi G. (2016). Blood Sampling Seasonality as an Important Preanalytical Factor for Assessment of Vitamin D Status. J. Med. Biochem..

[9] Papadakis G., Keramidas I., Kakava K., Pappa T., Villiotou V., Triantafillou E., Drosou A., Tertipi A., Kaltzidou V., Pappas A. (2015). Seasonal variation of serum vitamin D among Greek female patients with osteoporosis. In Vivo.

[10] Hypponen E., Power C. (2007). Hypovitaminosis D in British adults at age 45 y: Nationwide cohort study of dietary and lifestyle predictors. Am. J. Clin. Nutr..

[11] Kroll M.H., Bi C., Garber C.C., Kaufman H.W., Liu D., Caston-Balderrama A., Zhang K., Clarke N., Xie M., Reitz R.E. (2015). Temporal relationship between vitamin D status and parathyroid hormone in the United States. PLoS ONE.

[12] Lippi G., Montagnana M., Meschi T., Borghi L. (2012). Vitamin D concentration and deficiency across different ages and genders. Aging Clin. Exp. Res..

[13] Malyavskaya S., Kostrova G., Kudryavtsev A.V., Lebedev A. (2023). Low vitamin D levels among children and adolescents in an Arctic population. Scand. J. Public Health.

[14] Yesiltepe-Mutlu G., Aksu E.D., Bereket A., Hatun S. (2020). Vitamin D Status Across Age Groups in Turkey: Results of 108,742 Samples from a Single Laboratory. J. Clin. Res. Pediatr. Endocrinol..

[15] Dawson-Hughes B., Heaney R.P., Holick M.F., Lips P., Meunier P.J., Vieth R. (2005). Estimates of optimal vitamin D status. Osteoporos. Int..

[16] Smirnova D.V., Rehm C.D., Fritz R.D., Kutepova I.S., Soshina M.S., Berezhnaya Y.A. (2022). Vitamin D status of the Russian adult population from 2013 to 2018. Sci. Rep..

[17] Sarafin K., Durazo-Arvizu R., Tian L., Phinney K.W., Tai S., Camara J.E., Merkel J., Green E., Sempos C.T., Brooks S.P. (2015). Standardizing 25-hydroxyvitamin D values from the Canadian Health Measures Survey. Am. J. Clin. Nutr..

[18] Schleicher R.L., Sternberg M.R., Lacher D.A., Sempos C.T., Looker A.C., Durazo-Arvizu R.A., Yetley E.A., Chaudhary-Webb M., Maw K.L., Pfeiffer C.M. (2016). The vitamin D status of the US population from 1988 to 2010 using standardized serum concentrations of 25-hydroxyvitamin D shows recent modest increases. Am. J. Clin. Nutr..

[19] Rabenberg M., Scheidt-Nave C., Busch M.A., Thamm M., Rieckmann N., Durazo-Arvizu R.A., Dowling K.G., Skrabakova Z., Cashman K.D., Sempos C.T. (2018). Implications of standardization of serum 25-hydroxyvitamin D data for the evaluation of vitamin D status in Germany, including a temporal analysis. BMC Public Health.

[20] Hoge A., Donneau A.F., Streel S., Kolh P., Chapelle J.P., Albert A., Cavalier E., Guillaume M. (2015). Vitamin D deficiency is common among adults in Wallonia (Belgium, 51 degrees 30’ North): Findings from the Nutrition, Environment and Cardio-Vascular Health study. Nutr. Res..

[21] Hintzpeter B., Mensink G.B., Thierfelder W., Muller M.J., Scheidt-Nave C. (2008). Vitamin D status and health correlates among German adults. Eur. J. Clin. Nutr..

[22] Petrenya N., Lamberg-Allardt C., Melhus M., Broderstad A.R., Brustad M. (2020). Vitamin D status in a multi-ethnic population of northern Norway: The SAMINOR 2 Clinical Survey. Public Health Nutr..

[23] McKenna M.J., Murray B.F., O’Keane M., Kilbane M.T. (2015). Rising trend in vitamin D status from 1993 to 2013: Dual concerns for the future. Endocr. Connect..

[24] Nielsen N.O., Jorgensen M.E., Friis H., Melbye M., Soborg B., Jeppesen C., Lundqvist M., Cohen A., Hougaard D.M., Bjerregaard P. (2014). Decrease in vitamin D status in the Greenlandic adult population from 1987–2010. PLoS ONE.

[25] Ginde A.A., Liu M.C., Camargo C.A. (2009). Demographic differences and trends of vitamin D insufficiency in the US population, 1988–2004. Arch. Intern. Med..

[26] Herrick K.A., Storandt R.J., Afful J., Pfeiffer C.M., Schleicher R.L., Gahche J.J., Potischman N. (2019). Vitamin D status in the United States, 2011–2014. Am. J. Clin. Nutr..

[27] Kunz C., Hower J., Knoll A., Ritzenthaler K.L., Lamberti T. (2019). No improvement in vitamin D status in German infants and adolescents between 2009 and 2014 despite public recommendations to increase vitamin D intake in 2012. Eur. J. Nutr..

[28] Horvath L., Mirani S., Girgis M.M.F., Racz S., Bacskay I., Bhattoa H.P., Toth B.E. (2023). Six years’ experience and trends of serum 25-hydroxy vitamin D concentration and the effect of vitamin D_3_ consumption on these trends. Front. Pharmacol..

[29] Tuuminen T., Sorsa M., Tornudd M., Lahteenmaki P.L., Poussa T., Suonsivu P., Pitkanen E.M., Antila E., Jaakkola K. (2022). Long-term nutritional trends in the Finnish population estimated from a large laboratory database from 1987 to 2020. Sci. Rep..

[30] Bhattoa H.P., Konstantynowicz J., Laszcz N., Wojcik M., Pludowski P. (2017). Vitamin D: Musculoskeletal health. Rev. Endocr. Metab. Disord..

[31] Muscogiuri G., Annweiler C., Duval G., Karras S., Tirabassi G., Salvio G., Balercia G., Kimball S., Kotsa K., Mascitelli L. (2017). Vitamin D and cardiovascular disease: From atherosclerosis to myocardial infarction and stroke. Int. J. Cardiol..

[32] Munoz A., Grant W.B. (2022). Vitamin D and Cancer: An Historical Overview of the Epidemiology and Mechanisms. Nutrients.

[33] Grant W.B., Bhattoa H.P., Boucher B.J. (2017). Seasonal variations of U.S. mortality rates: Roles of solar ultraviolet-B doses, vitamin D, gene exp ression, and infections. J. Steroid Biochem. Mol. Biol..

[34] Grant W.B., Fakhoury H.M.A., Karras S.N., Al Anouti F., Bhattoa H.P. (2019). Variations in 25-Hydroxyvitamin D in Countries from the Middle East and Europe: The Roles of UVB Exposure and Diet. Nutrients.

[35] Holick M.F., Binkley N.C., Bischoff-Ferrari H.A., Gordon C.M., Hanley D.A., Heaney R.P., Murad M.H., Weaver C.M., Endocrine S. (2011). Evaluation, treatment, and prevention of vitamin D deficiency: An Endocrine Society clinical practice guideline. J. Clin. Endocrinol. Metab..

[36] Demay M.B., Pittas A.G., Bikle D.D., Diab D.L., Kiely M.E., Lazaretti-Castro M., Lips P., Mitchell D.M., Murad M.H., Powers S. (2024). Vitamin D for the Prevention of Disease: An Endocrine Society Clinical Practice Guideline. J. Clin. Endocrinol. Metab..

[37] Bischoff-Ferrari H.A., Giovannucci E., Willett W.C., Dietrich T., Dawson-Hughes B. (2006). Estimation of optimal serum concentrations of 25-hydroxyvitamin D for multiple health outcomes. Am. J. Clin. Nutr..

[38] Priemel M., von Domarus C., Klatte T.O., Kessler S., Schlie J., Meier S., Proksch N., Pastor F., Netter C., Streichert T. (2010). Bone mineralization defects and vitamin D deficiency: Histomorphometric analysis of iliac crest bone biopsies and circulating 25-hydroxyvitamin D in 675 patients. J. Bone Miner. Res..

[39] Dobnig H., Pilz S., Scharnagl H., Renner W., Seelhorst U., Wellnitz B., Kinkeldei J., Boehm B.O., Weihrauch G., Maerz W. (2008). Independent association of low serum 25-hydroxyvitamin d and 1,25-dihydroxyvitamin d levels with all-cause and cardiovascular mortality. Arch. Intern. Med..

[40] Herrmann M., Sullivan D.R., Veillard A.S., McCorquodale T., Straub I.R., Scott R., Laakso M., Topliss D., Jenkins A.J., Blankenberg S. (2015). Serum 25-hydroxyvitamin D: A predictor of macrovascular and microvascular complications in patients with type 2 diabetes. Diabetes Care.

[41] Grant W.B., Boucher B.J., Cheng R.Z., Pludowski P., Wimalawansa S.J. (2025). Vitamin D and Cardiovascular Health: A Narrative Review of Risk Reduction Evidence. Nutrients.

[42] Grant W.B., Wimalawansa S.J., Pludowski P., Cheng R.Z. (2025). Vitamin D: Evidence-Based Health Benefits and Recommendations for Population Guidelines. Nutrients.

[43] Holick M.F. (2009). Vitamin D Status: Measurement, Interpretation, and Clinical Application. Ann. Epidemiol..

[44] Woitge H.W., Knothe A., Witte K., Schmidt-Gayk H., Ziegler R., Lemmer B., Seibel M.J. (2000). Circaannual rhythms and interactions of vitamin D metabolites, parathyroid hormone, and biochemical markers of skeletal homeostasis: A prospective study. J. Bone Miner. Res..

[45] Schottker B., Jorde R., Peasey A., Thorand B., Jansen E.H., Groot L., Streppel M., Gardiner J., Ordonez-Mena J.M., Perna L. (2014). Vitamin D and mortality: Meta-analysis of individual participant data from a large consortium of cohort studies from Europe and the United States. BMJ.

[46] Zittermann A., Ernst J.B., Gummert J.F., Borgermann J. (2014). Vitamin D supplementation, body weight and human serum 25-hydroxyvitamin D response: A systematic review. Eur. J. Nutr..

[47] Glendenning P., Chew G.T., Seymour H.M., Gillett M.J., Goldswain P.R., Inderjeeth C.A., Vasikaran S.D., Taranto M., Musk A.A., Fraser W.D. (2009). Serum 25-hydroxyvitamin D levels in vitamin D-insufficient hip fracture patients after supplementation with ergocalciferol and cholecalciferol. Bone.

[48] Wamberg L., Pedersen S.B., Richelsen B., Rejnmark L. (2013). The effect of high-dose vitamin D supplementation on calciotropic hormones and bone mineral density in obese subjects with low levels of circulating 25-hydroxyvitamin d: Results from a randomized controlled study. Calcif. Tissue Int..

[49] Diamond T., Wong Y.K., Golombick T. (2013). Effect of oral cholecalciferol 2,000 versus 5,000 IU on serum vitamin D, PTH, bone and muscle strength in patients with vitamin D deficiency. Osteoporos. Int..

[50] Rybchyn M.S., Abboud M., Puglisi D.A., Gordon-Thomson C., Brennan-Speranza T.C., Mason R.S., Fraser D.R. (2020). Skeletal Muscle and the Maintenance of Vitamin D Status. Nutrients.

[51] Mason R.S., Rybchyn M.S., Abboud M., Brennan-Speranza T.C., Fraser D.R. (2019). The Role of Skeletal Muscle in Maintaining Vitamin D Status in Winter. Curr. Dev. Nutr..

[52] Hollis B.W. (2004). Editorial: The determination of circulating 25-hydroxyvitamin D: No easy task. J. Clin. Endocrinol. Metab..

[53] Makris K., Bhattoa H.P., Cavalier E., Phinney K., Sempos C.T., Ulmer C.Z., Vasikaran S.D., Vesper H., Heijboer A.C. (2021). Recommendations on the measurement and the clinical use of vitamin D metabolites and vitamin D binding protein—A position paper from the IFCC Committee on bone metabolism. Clin. Chim. Acta.

[54] Moreau E., Bacher S., Mery S., Le Goff C., Piga N., Vogeser M., Hausmann M., Cavalier E. (2016). Performance characteristics of the VIDAS(R) 25-OH Vitamin D Total assay-comparison with four immunoassays and two liquid chromatography-tandem mass spectrometry methods in a multicentric study. Clin. Chem. Lab. Med..

[55] Depreter B., Heijboer A.C., Langlois M.R. (2013). Accuracy of three automated 25-hydroxyvitamin D assays in hemodialysis patients. Clin. Chim. Acta.

[56] Heijboer A.C., Blankenstein M.A., Kema I.P., Buijs M.M. (2012). Accuracy of 6 routine 25-hydroxyvitamin D assays: Influence of vitamin D binding protein concentration. Clin. Chem..

[57] Cavalier E., Lukas P., Crine Y., Peeters S., Carlisi A., Le Goff C., Gadisseur R., Delanaye P., Souberbielle J.C. (2014). Evaluation of automated immunoassays for 25(OH)-vitamin D determination in different critical populations before and after standardization of the assays. Clin. Chim. Acta.

[58] Binkley N., Sempos C.T., Vitamin D.S.P. (2014). Standardizing vitamin D assays: The way forward. J. Bone Miner. Res..

[59] Sempos C.T., Durazo-Arvizu R.A., Binkley N., Jones J., Merkel J.M., Carter G.D. (2016). Developing vitamin D dietary guidelines and the lack of 25-hydroxyvitamin D assay standardization: The ever-present past. J. Steroid Biochem. Mol. Biol..

[60] Cashman K.D., Dowling K.G., Skrabakova Z., Kiely M., Lamberg-Allardt C., Durazo-Arvizu R.A., Sempos C.T., Koskinen S., Lundqvist A., Sundvall J. (2015). Standardizing serum 25-hydroxyvitamin D data from four Nordic population samples using the Vitamin D Standardization Program protocols: Shedding new light on vitamin D status in Nordic individuals. Scand. J. Clin. Lab. Investig..

[61] Cashman K.D., Kiely M., Kinsella M., Durazo-Arvizu R.A., Tian L., Zhang Y., Lucey A., Flynn A., Gibney M.J., Vesper H.W. (2013). Evaluation of Vitamin D Standardization Program protocols for standardizing serum 25-hydroxyvitamin D data: A case study of the program’s potential for national nutrition and health surveys. Am. J. Clin. Nutr..

[62] Jakab E., Kalina E., Petho Z., Pap Z., Balogh A., Grant W.B., Bhattoa H.P. (2017). Standardizing 25-hydroxyvitamin D data from the HunMen cohort. Osteoporos. Int..

[63] Altieri B., Cavalier E., Bhattoa H.P., Perez-Lopez F.R., Lopez-Baena M.T., Perez-Roncero G.R., Chedraui P., Annweiler C., Della Casa S., Zelzer S. (2020). Vitamin D testing: Advantages and limits of the current assays. Eur. J. Clin. Nutr..

